# Supplementary material for: The influence of rhizosphere microbial diversity on the accumulation of active compounds in farmed Scutellaria baicalensis
Source: PeerJ. 2024 Dec 24;12:e18749. doi: 10.7717/peerj.18749 (PMC11674151; doi:10.7717/peerj.18749)
Supplement: Supplemental Information 1 [file peerj-12-18749-s001.docx]

Content of active compounds of *Scutellaria baicalensis*

|  | baicalin（%） | oroxindin（%） | baicalein（%） | wogonin（%） | oroxylin A（%） |
| --- | --- | --- | --- | --- | --- |
| KC | 9.09 | 2.71 | 5.21 | 0.23 | 0.51 |
| KC | 9.21 | 2.66 | 5.18 | 0.23 | 0.51 |
| KC | 9.17 | 2.61 | 5.22 | 0.2 | 0.48 |
| PQ | 13.85 | 2.7 | 2.66 | 0.43 | 0.12 |
| PQ | 14.56 | 2.84 | 2.8 | 0.45 | 0.12 |
| PQ | 14.1 | 2.75 | 2.71 | 0.43 | 0.12 |
| FN | 10.32 | 2.41 | 2.11 | 0.31 | 0.12 |
| FN | 10.28 | 2.44 | 2.14 | 0.33 | 0.12 |
| FN | 10.31 | 2.4 | 2.12 | 0.3 | 0.11 |

Note: here is the raw date.
