# Supplementary material for: The influence of rhizosphere microbial diversity on the accumulation of active compounds in farmed Scutellaria baicalensis
Source: PeerJ. 2024 Dec 24;12:e18749. doi: 10.7717/peerj.18749 (PMC11674151; doi:10.7717/peerj.18749)
Supplement: Supplemental Information 2 [file peerj-12-18749-s002.docx]

Physicochemistry of the rhizosphere soil of cultivated *Scutellaria baicalensis*

| number | TN （g/kg) | TP （g/kg) | TK （g/kg) | OM （g/kg) | HN （mg/kg) | AP （mg/kg) | AK （mg/kg) |
| --- | --- | --- | --- | --- | --- | --- | --- |
| KC | 1.11 | 0.97 | 19.36 | 20.0 | 79.8 | 41.2 | 120 |
| KC | 1.10 | 0.95 | 19.55 | 19.8 | 78.8 | 40.8 | 123 |
| KC | 1.13 | 0.96 | 19.46 | 19.6 | 77.0 | 42.0 | 125 |
| PQ | 0.92 | 0.58 | 20.99 | 16.0 | 67.2 | 13.1 | 209 |
| PQ | 0.94 | 0.57 | 20.80 | 16.4 | 68.3 | 13.4 | 205 |
| PQ | 0.93 | 0.58 | 20.87 | 16.5 | 69.3 | 13.3 | 207 |
| FN | 0.83 | 0.70 | 18.98 | 12.5 | 67.9 | 3.5 | 141 |
| FN | 0.82 | 0.69 | 19.18 | 12.4 | 66.5 | 3.4 | 140 |
| FN | 0.83 | 0.71 | 19.09 | 12.1 | 68.6 | 3.5 | 143 |
| number | AF （mg/kg） | AMn  （mg/kg） | ACu  （mg/kg） | AZn  （mg/kg） | ECa  （g/kg） | EMg  （g/kg） | pH |
| KC | 11.6 | 8.5 | 1.63 | 2.28 | 4.00 | 0.37 | 7.37 |
| KC | 11.7 | 8.6 | 1.64 | 2.30 | 3.96 | 0.38 | 7.41 |
| KC | 11.4 | 8.4 | 1.59 | 2.23 | 4.01 | 0.38 | 7.45 |
| PQ | 9.1 | 7.6 | 1.12 | 0.78 | 6.37 | 0.34 | 7.83 |
| PQ | 9.6 | 8.0 | 1.17 | 0.79 | 6.31 | 0.35 | 7.78 |
| PQ | 9.3 | 7.8 | 1.14 | 0.80 | 6.40 | 0.34 | 7.80 |
| FN | 8.0 | 7.6 | 0.54 | 1.08 | 8.78 | 0.17 | 7.96 |
| FN | 8.4 | 8.0 | 0.56 | 1.14 | 8.71 | 0.18 | 7.89 |
| FN | 8.1 | 7.7 | 0.54 | 1.10 | 8.81 | 0.17 | 7.85 |

Note: here is the raw date.
