# Supplementary material for: The influence of rhizosphere microbial diversity on the accumulation of active compounds in farmed Scutellaria baicalensis
Source: PeerJ. 2024 Dec 24;12:e18749. doi: 10.7717/peerj.18749 (PMC11674151; doi:10.7717/peerj.18749)
Supplement: Supplemental Information 3 [file peerj-12-18749-s003.docx]

S 1. Relative abundance of dominant bacterial genera

| Dominant bacterial genera | KC | PQ | FN |
| --- | --- | --- | --- |
| *norank_f__Vicinamibacteraceae* | 7.07% | 6.55% | 7.00% |
| *norank_f__norank_o__Vicinamibacterales* | 5.72% | 4.39% | 4.83% |
| *RB41* | 5.14% | 4.31% | 4.91% |
| *norank_f__JG30-KF-CM45* | 2.97% | 4.89% | 3.67% |
| *Arthrobacter* | 2.40% | 5.70% | 1.61% |
| *norank_f__Gemmatimonadaceae* | 2.44% | 2.81% | 4.29% |
| *norank_f__norank_o__norank_c__MB-A2-108* | 2.39% | 1.99% | 3.98% |
| *norank_f__norank_o__norank_c__KD4-96* | 2.48% | 2.36% | 3.07% |
| *Bacillus* | 2.10% | 1.21% | 1.68% |
| *MND1* | 2.05% | 1.08% | 1.79% |
| *norank_f__norank_o__Rokubacteriales* | 1.79% | 1.12% | 1.98% |
| *Rubrobacter* | 1.04% | 1.86% | 1.74% |
| *unclassified_k__norank_d__Bacteria* | 1.24% | 1.08% | 1.79% |
| *Gaiella* | 1.14% | 1.38% | 1.56% |
| *Microvirga* | 1.17% | 1.85% | 1.01% |
| *Sphingomonas* | 0.96% | 1.89% | 0.79% |
| *norank_f__norank_o__Gaiellales* | 1.07% | 1.41% | 1.11% |
| *norank_f__67-14* | 1.00% | 1.05% | 1.44% |
| *Blastococcus* | 1.00% | 1.54% | 0.87% |
| *norank_f__Xanthobacteraceae* | 0.94% | 0.79% | 1.33% |
| *norank_f__Roseiflexaceae* | 0.94% | 1.02% | 1.09% |
| *Nocardioides* | 0.94% | 1.22% | 0.70% |
| *norank_f__norank_o__norank_c__Gitt-GS-136* | 1.00% | 0.60% | 1.10% |
| *Ellin6055* | 0.63% | 1.14% | 0.81% |
| *norank_f__norank_o__Subgroup_17* | 0.91% | 0.44% | 1.04% |
| *unclassified_f__Micromonosporaceae* | 0.45% | 1.11% | 0.63% |
| *norank_f__norank_o__norank_c__bacteriap25* | 1.05% | 0.46% | 0.69% |
| *norank_f__norank_o__0319-7L14* | 0.14% | 0.06% | 1.04% |
| others | 47.85% | 44.70% | 42.45% |
