# Supplementary material for: The influence of rhizosphere microbial diversity on the accumulation of active compounds in farmed Scutellaria baicalensis
Source: PeerJ. 2024 Dec 24;12:e18749. doi: 10.7717/peerj.18749 (PMC11674151; doi:10.7717/peerj.18749)
Supplement: Supplemental Information 4 [file peerj-12-18749-s004.docx]

S 2. Relative abundance of dominant fungal genera

| dominant fungal genus | KC | PQ | FN |
| --- | --- | --- | --- |
| *Metarhizium* | 0.73% | 1.06% | 26.81% |
| *Mortierella* | 12.05% | 3.02% | 10.00% |
| *Cladosporium* | 5.03% | 13.72% | 3.91% |
| *Alternaria* | 4.55% | 13.11% | 1.77% |
| *Paraphoma* | 6.61% | 4.21% | 5.38% |
| *Epicoccum* | 1.53% | 5.19% | 0.82% |
| *unclassified_p__Ascomycota* | 1.61% | 2.23% | 2.81% |
| *unclassified_k__Fungi* | 1.55% | 0.84% | 3.43% |
| *Solicoccozyma* | 3.52% | 0.69% | 1.31% |
| *Gibberella* | 2.17% | 2.10% | 1.02% |
| *Fusarium* | 2.07% | 0.78% | 1.91% |
| *unclassified_o__Glomerellales* | 1.68% | 1.06% | 1.97% |
| *Chaetomium* | 2.70% | 0.62% | 0.71% |
| *Neocosmospora* | 1.84% | 0.84% | 1.04% |
| *Tausonia* | 3.44% | 0.14% | 0.03% |
| *Titaea* | 2.04% | 0.26% | 1.19% |
| *Didymella* | 0.94% | 1.83% | 0.70% |
| *Minimedusa* | 3.07% | 0.11% | 0.26% |
| *unclassified_o__Sordariales* | 3.07% | 0.07% | 0.06% |
| *Cercospora* | 0.17% | 3.01% | 0.00% |
| *Talaromyces* | 2.70% | 0.27% | 0.20% |
| *Periconia* | 0.55% | 1.69% | 0.66% |
| *Vishniacozyma* | 0.50% | 1.69% | 0.68% |
| *unclassified_o__Helotiales* | 0.13% | 2.03% | 0.68% |
| *Botrytis* | 0.53% | 2.12% | 0.04% |
| *Articulospora* | 0.50% | 0.73% | 1.15% |
| *Phaeosphaeria* | 0.26% | 1.90% | 0.06% |
| *Coprinellus* | 0.03% | 0.03% | 2.14% |
| *Spegazzinia* | 0.00% | 1.91% | 0.00% |
| *unclassified_f__Glomeraceae* | 1.54% | 0.05% | 0.18% |
| *unclassified_f__Arthopyreniaceae* | 1.31% | 0.25% | 0.18% |
| *Hannaella* | 0.26% | 1.20% | 0.25% |
| *Coniothyrium* | 0.15% | 1.46% | 0.08% |
| *Phaeomycocentrospora* | 0.18% | 0.00% | 1.40% |
| *unclassified_f__Sporormiaceae* | 1.23% | 0.18% | 0.17% |
| *Cyphellophora* | 0.32% | 1.03% | 0.15% |
| *Boeremia* | 0.03% | 1.16% | 0.26% |
| *Isaria* | 0.11% | 0.00% | 1.32% |
| *Dioszegia* | 0.16% | 1.17% | 0.10% |
| *unclassified_f__Phaeosphaeriaceae* | 0.02% | 1.27% | 0.07% |
| *Monodictys* | 1.27% | 0.02% | 0.06% |
| *Inocybe* | 0.00% | 0.00% | 1.18% |
| others | 27.86% | 24.93% | 23.84% |
